# RBM19 is essential for preimplantation development in the mouse

**DOI:** 10.1186/1471-213X-8-115

**Published:** 2008-12-16

**Authors:** Jian Zhang, Amber J Tomasini, Alan N Mayer

**Affiliations:** 1 Division of Gastroenterology, Department of Pediatrics, Medical College of Wisconsin and Children's Research Institute, Milwaukee, WI 53226, USA

## Abstract

**Background:**

RNA-binding motif protein 19 (RBM19, NCBI Accession # NP_083038) is a conserved nucleolar protein containing 6 conserved RNA recognition motifs. Its biochemical function is to process rRNA for ribosome biogenesis, and it has been shown to play a role in digestive organ development in zebrafish. Here we analyzed the role of RBM19 during mouse embryonic development by generating mice containing a mutation in the *Rbm19 *locus via gene-trap insertion.

**Results:**

Homozygous mutant embryos failed to develop beyond the morula stage, showing defective nucleologenesis, activation of apoptosis, and upregulation of P53 target genes. A unique feature of RBM19 is its localization to the cytoplasm in morula stage-embryos, whereas most other nucleolar proteins are localized to the nucleolar precursor body (NPB). The nucleoli in the *Rbm19 *mutant embryos remain immature, yet they can carry out rRNA synthesis. The timing of developmental arrest occurs after expression of the inner cell mass markers OCT3/4 and NANOG, but prior to the specification of trophectoderm as reflected by CDX2 expression.

**Conclusion:**

The data indicate that RBM19 is essential for preimplantation development, highlighting the importance of de novo nucleologenesis during this critical developmental stage.

## Background

During early embryogenesis, the zygotic nucleolus assembles from a ground state following the disassembly of the maternal nucleolus after pronuclear fusion [1]. "De novo" nucleologenesis begins in the mouse at the end of the 2^nd ^cell cycle with the initiation of ribosomal RNA transcription. An early morphological intermediate of the reforming nucleolus is the nucleolus precursor body (NPB) [2]. The NPB can be detected through the morula stage, after which it progressively adopts the classic tripartite morphology as seen in the blastocyst and thereafter. Ribosomal RNA transcription begins on the surface of the sphere, where the nucleolar organizing regions (NORs) of the genome are located [3]. In contrast to somatic (i.e. post-mitotic) nucleologenesis, the mechanism and role in development of de novo nucleologenesis are not well understood.

The role of nucleolar proteins during early mouse development has been investigated by gene targeting in the mouse. In virtually all cases, genetic ablation of protein function results in developmental arrest prior to birth, but at stages that might not be predictable *a priori *based on the encoded protein's function. For example, in embryos mutant for the proteins involved in ribosomal (r)RNA synthesis or processing, such as pescadillo-1 (PES-1) [4], fibrillarin [5], RNA polymerase 1–2 [6] and Surf6 [7], arrest occurs at the morula stage. However, in the *tif1a *mutant, which ablates a protein mediating the earlier process of rRNA transcription, arrest occurs after implantation [8]. Knockout of B23/nucleophosmin, which is required for rRNA processing and also chromosome segregation, arrested development between E9.5 and E12.5 [9]. A unifying feature in all these mutant embryos was disruption of the nucleolus and increased apoptosis. It remains unclear to what extent mutant phenotypes arise from a shortage of ribosomes or from stress signals originating from a dysfunctional nucleolus.

Here we studied the role of RBM19 during early mouse embryonic development. RBM19 is a conserved nucleolar protein distinguished by its 6 RNA recognition motifs (RRM) [10-12]. It has been implicated in processing pre-ribosomal RNA [11]. It was also found to be essential for digestive organ development in the zebrafish in two independent genetic screens [12,13], and is expressed preferentially in the progenitor compartment of the mammalian intestine [14]. To assess its function during mouse development, we generated a mutant containing a gene-trap insertion into the *Rbm19 *locus. As described below, we found that RBM19 is required for preimplantation development.

## Results

### Molecular characterization of the *Rbm19*^*XC768 *^mutation

**Figure 1:**
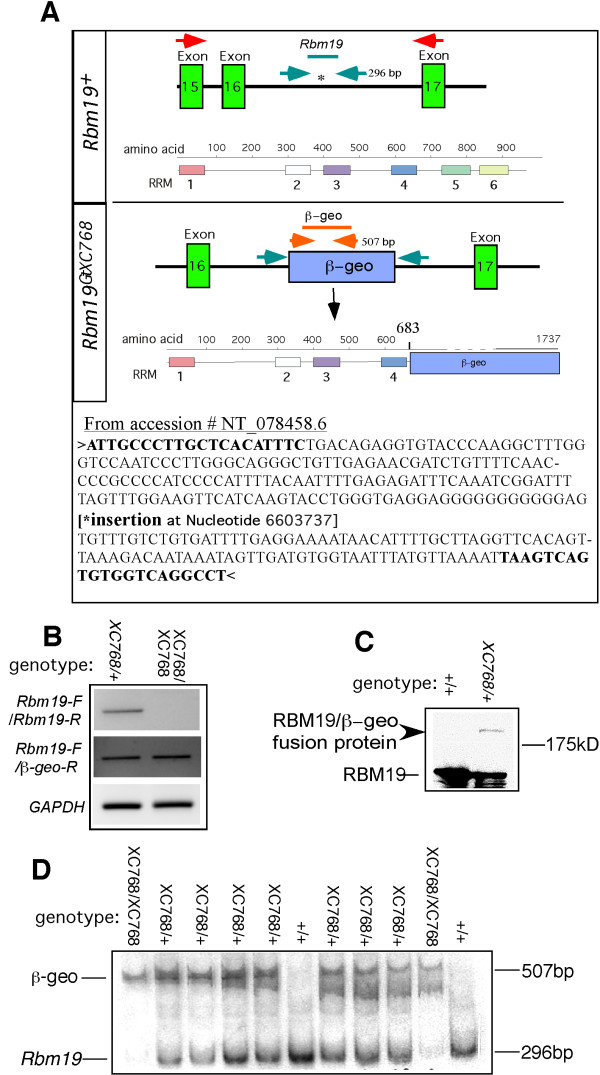
***Rbm19*^*GtXC768*/*GtXC768 *^gene-trap characterization**. (A) The insertion site of the gene trap vector is in intron 16 (*), resulting in loss of RRMs 5 and 6, along with the addition of a 116 kD β-geo protein. The exact insertion site is shown by the sequences flanking the gene trap insertion. The nucleotide number corresponds to the sequence available at NCBI accession NT_078458.6. The bold nucleotides represent the primer sequences we used to detect the wild-type *Rbm19 *allele. (B) RT-PCR shows no detectable *Rbm19*^+ ^transcript in the mutant E3.5 embryos using *Rbm19*-specific primers made to exons flanking the insertion site (red arrows, panel A). The expected product was detected when using an *Rbm19*-specific forward primer and β-geo reverse primer in the mutant embryos. (C) Western blot for RBM19 (amino-terminal epitope [14]) shows the expected 191 kD fusion protein band in the heterozygous mouse intestine (arrow), but not in homozygous wild-type. (D) ^32^P-labeled PCR genotyping of E3.5 embryos from *Rbm19*^*GtXC768*/+ ^intercrosses. The embryos were genotyped by a duplex PCR strategy. *Rbm19*-specific primers amplify a 296-bp DNA fragment across the insertion site (panel A, WT), which is disrupted by the genetrap vector in the mutant. β-geo-specific primers amplify a 507-bp fragment from the gene trap vector (panel B, *Rbm19*^*GtXC768*^.)

We identified an ES cell line containing a gene-trap insertion in the *Rbm19 *locus *Rbm19*^*Gt(pGT1Lxf)XC768 *^(*Rbm19*^*GtXC768*^) by searching the BayGenomics database [15]. The insertion is within intron 16 and predicted to create a transcript that joins the sequences from exon 16 to the β-galactosidase open reading frame. The exact insertion site was determined by empirically testing a series of PCR primer pairs consisting of one *Rbm19*-specific primer and one primer to various sites in the gene trap vector. PCR products were sequenced, enabling detection of the insertion site. The gene trap message should encode a fusion protein in which the two C-terminal RRM domains are omitted and substituted with the β-galactosidase-neomycin resistance protein, leaving the four N-terminal RRM domains intact (Figure 1A). In heterozygotes, by quantitative PCR we detected 5 copies of the insertion vector. These animals had been outcrossed at least 4 times, yielding 50% heterozygous offspring, indicating that the multiple copies are tightly linked. The most likely explanation is that multiple copies were integrated in tandem, which is known to occur with plasmid vectors such as pGT1Lxf when electroporated into ES cells [16]. Since it is formally possible that the integrated DNA could get excised during splicing, we characterized the transcripts produced in homozygous vs. heterozygote embryos by reverse transcriptase (RT)-PCR. Using *Rbm19*-specific primers to exons flanking the insertion site or an *Rbm19*-specific forward primer paired with a β-geo reverse primer, we could detect both wild-type and mutant-specific bands, respectively. We detected both bands in the heterozygotes, but only the mutant band in homozygote-derived cDNA.

To further demonstrate that the β-geo insertion indeed generated a fusion protein we performed a Western blot on extract from duodenum of *Rbm19*^*GtXC768*/+ ^adult mice, using anti-RBM19 antibody directed against an epitope near the amino terminal of the protein [14]. As expected, we detected a distinct, albeit faint, band corresponding to the predicted size of the RBM19-β geo fusion protein (191 kilodaltons (kD)) in the heterozygote but not in the wild-type extract (Figure 1C). We could not perform Western blotting on homozygous mutant embryos due to arrest at the morula stage (see below) and consequent lack of adequate material.

We fine-mapped the insertion site to single nucleotide resolution using a series of PCR primers complementary to mouse genomic sequences distributed across intron 16. Sequencing the PCR products allowed us to identify the junctions between gene-trap vector and genomic DNA. We did not detect any deletions. We were then able to perform allele-specific multiplex PCR for all subsequent genotyping (Figure 1D).

### Characterization of the *Rbm19*^*GtXC768 *^mutant phenotype

**Figure 2:**
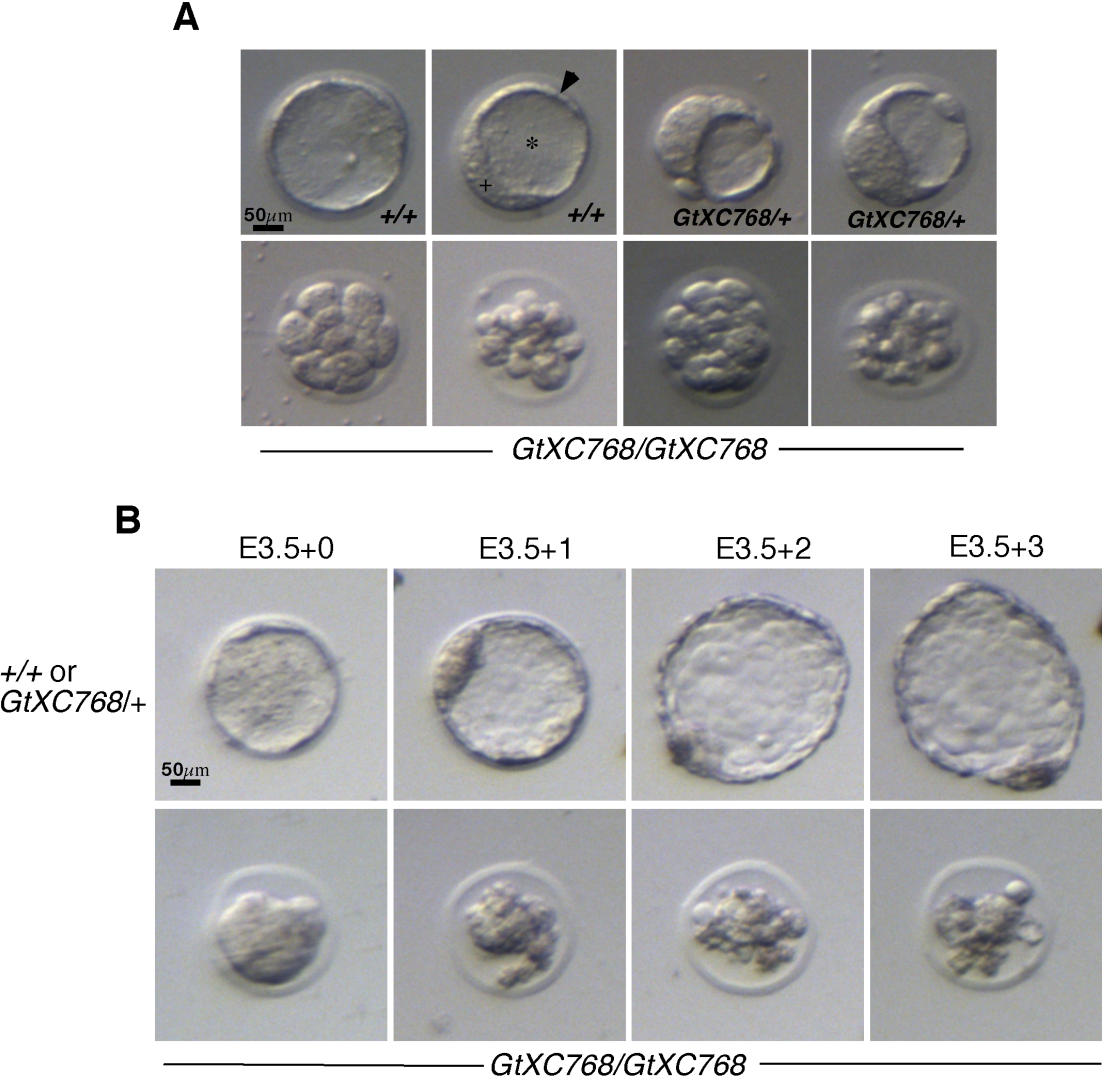
***Rbm19*^*GtXC768*/*GtXC768 *^embryos undergo morula arrest**. (A) Live stereoscope images of *Rbm19*^*GtXC768*/+^, *Rbm19*^+/+ ^or *Rbm19*^*GtXC768*/*GtXC768 *^E3.5 embryos (confirmed subsequently by genotyping). Homozygous mutants fail to form an inner cell mass (+), trophectoderm cell layer (arrow head), and blastocoel cavity (*). (B) Live images of embryos cultured in vitro. Embryos derived from *Rbm19*^*GtXC768*/+ ^intercrosses were explanted at E3.5 (day 0), and grown in KSOM for the indicated number of days. Approximately 25% of the embryos arrested in the morula stage; the others hatched from the zona pellucida (Day 2) and continued to develop.

We generated chimeric mice by blastocyst injection, and bred these to obtain founder *Rbm19*^*GtXC768*/+ ^mice. The progeny of heterozygote outcrosses were in the expected Mendelian ratio (50%, n > 300) and the heterozygote mice displayed no discernible mutant phenotype, surviving beyond 2 years without overt pathology. Interbreeding the *Rbm19*^*GtXC768*/+ ^mice yielded neither live births nor embryos prior to E6.5 that were homozygous. At E3.5 the intercrosses consistently yielded a mixture of morula and blastocyst-stage embryos (Table 1). Genotyping of these embryos demonstrated that the morula-stage embryos were homozygote mutants, whereas the blastocyst stage embryos were uniformly *Rbm19*^+/+ ^or *Rbm19*^*GtXC768*/+ ^(Figure 2A). Visual screening at E3.5 was 93% predictive for genotype (n = 70). Prior to E3.5, intercrosses yielded early stage embryos that were indistinguishable from each other, despite the presence of homozygote mutants among these progeny. To discern if the mutant embryos at E3.5 were arrested at this stage or simply delayed, we cultured the embryos. Normal-appearing E3.5 embryos formed expanded blastocysts, but the E3.5 morula-stage embryos remained arrested at the morula stage for an additional day, and then degenerated (Figure 2B). Thus the *Rbm19*^*GtXC768*/*GtXC768 *^embryos arrest prior to forming the blastocyst. No wild-type intercrosses in the C57/BL6 background yielded embryos similarly delayed at E3.5.

**Table 1:** Genotypes of the offspring and embryos from *Rbm19*^*GtXC768*/+ ^intercrosses

**Stage**	**Number of embryos (%)**			
	
	**+/+**	**+/-**	**-/-**	**ND**
E3.5	11(16%)	41(58%)	13(19%)	5(7%)

E6.5	2(33%)	4(67%)	0	0

E7.5	5(45%)	6(55%)	0	0

E8.5	4(40%)	5(50%)	0	1(10%)

E9.5	4(50%)	4(50%)	0	0

E10.5	5(71%)	2(29%)	0	0

E11.5	1(33%)	2(67%)	0	0

E12.5	2(67%)	1(33%)	0	0

3 Week	17(30%)	40(70%)	0	0

E6.5-3 weeks	40	64	0	1

### RBM19 localization during preimplantation development

**Figure 3:**
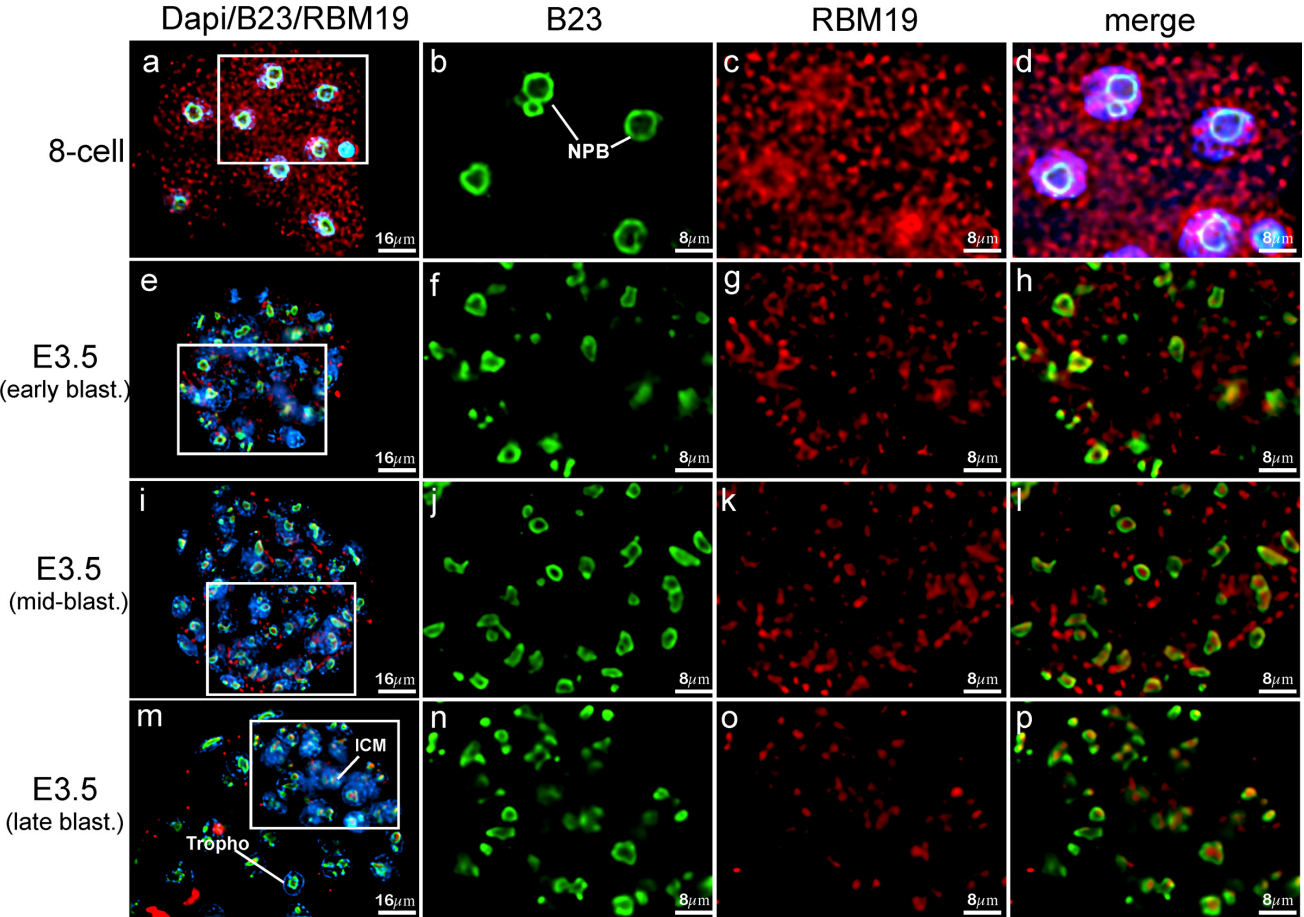
**RBM19 is expressed in a dynamic pattern during preimplantation development**. (a-d) RBM19 immunostaining in the 8-cell stage embryos localizes to cytoplasmic granules. In contrast, B23 is localized around the NPB. (e-h) In the early blastocyst, RBM19 begins to co-localize with B23 within nuclear foci, but is also noted in the cytoplasm. (i-l) In the mid-blastocyst, the inner cell mass (ICM) is becoming more distinct (oriented toward 5 o'clock) and RBM19 continues to co-localize increasingly with B23. RBM19 is retained in the cytoplasm to a greater extent in the cells that are located in the presumptive ICM. (M-P) In the late blastocyst the ICM is easily identified oriented toward 2 o'clock. The localization of RBM19 is mostly concordant with B23 thereafter.

To understand how the RBM19 protein might function during early development, we determined its subcellular localization by immunofluorescence. At the 4–8 cell stages we found RBM19 localized to discrete foci distributed throughout the cytoplasm and nucleoplasm (Figure 3). This result was surprising, since other nucleolar proteins such as nucleolin, fibrillarin [17], NOPP140 [18] and pescadillo-1 (PES-1) [4] localize to the periphery of the NPB in cleavage and morula stage embryos. Double staining with B23 confirmed that prominent NPBs can be visualized at this stage, but most of the RBM19 does not localize to these structures. As the blastocyst forms, RBM19 becomes increasingly localized to the nucleolus and less to the cytoplasm. By the late blastocyst stage, when nucleologenesis is largely completed, we observed only a few cytoplasmic RBM19-positive foci, with most of the RBM19 protein seen in the nucleolus.

Since RBM19 contains 6 RRMs and has been shown to bind RNA [12,19], we considered the possibility that RBM19 localizes to P-bodies, which store and process maternal RNA in the oocyte [20]. We performed double immunofluorescence staining for RBM19 and the P-body-specific protein GW182, and found that they do not colocalize (Additional file 1), indicating that RBM19-positive cytoplasmic foci and P-bodies are distinct structures.

### Nucleologenesis is disrupted in *Rbm19*^*GtXC768/Gt XC768 *^mutant embryos

**Figure 4:**
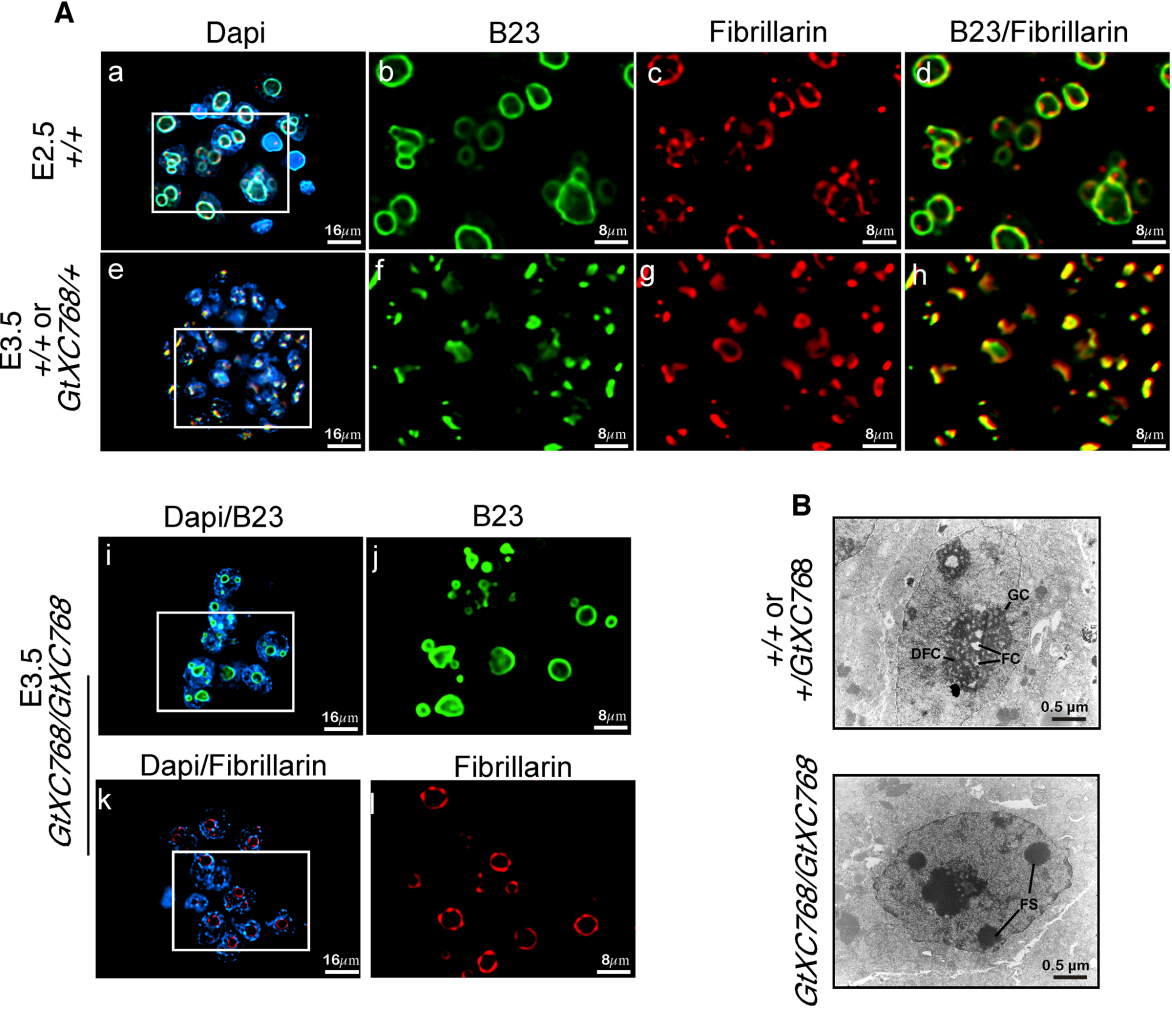
**Nucleologenesis in the *Rbm19*^*GtXC768*/*GtXC768 *^mutant embryos**. (A) Embryos from an *Rbm19*^*GtXC768*/+ ^intercross were sorted based on appearance and designated either wild-type-appearing (*Rbm19*^*GtXC768*/+ ^or *Rbm19*^+/+^) or *Rbm19*^*GtXC768*/*GtXC768*^mutants. Embryos at the indicated stages were stained with anti-B23 (green) and anti-fibrillarin (red) antibodies. (a-d) B23 and fibrillarin localization reveals the morphology of the NPB in E2.5 wild-type embryos. (e-h) At E3.5 nucleoli are smaller and more numerous than earlier stage shown in panels a-d (i-j) and (k-l). In the *Rbm19*^*GtXC768*/*GtXC768 *^mutant, B23 and fibrillarin, respectively, show a heterogeneous pattern, mostly resembling NPBs as seen in panel b, but with a few smaller foci suggesting partial nucleolar differentiation. (B) Electron micrograph of E3.5 wild type and *Rbm19*^*GtXC768*/*GtXC768 *^mutant embryos. Wild type embryos have mature nucleoli with differentiated nucleolar regions including fibrillar components (*FC*), dense fibrillar components (*DFC*), and granular components (*GC*). Mutant blastomeres (5 embryos examined) contained multiple fibrillar spheres (FS) that were presumed to be the nucleolar precursor bodies, but failed to form well-differentiated nucleoli.

Between the morula and blastocyst stages, the NPB undergoes a morphogenic transition to a mature, tripartite nucleolus [2]. As shown in Figure 3, this transition coincides with the relocalization of RBM19 from cytoplasm to nucleolus, suggesting that these two processes may be linked. To test if RBM19 function may be required to form the mature nucleolus, we performed immunostaining for the nucleolar markers B23 and fibrillarin in the *Rbm19*^*GtXC768*/*GtXC768 *^embryos (Figure 4A). The predominant B23 and fibrillarin staining patterns in the mutant embryos resemble that of the wild-type morula. In most cases we saw B23 and fibrillarin localized to large rings, consistent with the periphery of NPBs. To further characterize nucleologenesis in the *Rbm19*^*GtXC768*/*GtXC768 *^mutants we examined the embryos using transmission electron microscopy (Figure 4B). The mutant E3.5 embryos lack the mature, tripartite nucleoli clearly evident in wild-type blastocysts. Instead, the mutants contain spheres resembling NPBs, indicating arrest of nucleologenesis.

Since the nucleolus is generated by the collective action of the ribosome biogenesis apparatus, we also assessed whether rRNA transcription might be impaired in the *Rbm19*^*GtXC768*/*GtXC768 *^mutant. We measured BrUTP incorporation in cultured embryos by double immunofluorescence staining with anti-BrdU and anti-fibrillarin antibodies (Additional file 2). We found that both wild-type and mutant embryos incorporated BrUTP that colocalized with fibrillarin. This indicated that RBM19 is not required for the assembly of the rRNA transcription apparatus on the surface of the NPB.

### Timing of developmental arrest in *Rbm19*^*GtXC768/GtXC768 *^mutant embryos

A feature of the *Rbm19*^*GtXC768*/*GtXC768 *^mutant phenotype is failure of the morula to undergo compaction (Figure 2). In the mutant, the margins between individual blastomeres can still be appreciated at the 16-cell stage, by which time compaction has already occurred in wild-type embryos. E-cadherin is a cell adhesion molecule essential for compaction of the morula [21]. To test if E-cadherin is expressed and properly localized in the mutant embryos, we performed whole mount immunofluorescence staining. E-cadherin was present and appeared to be normally localized between the blastomeres (Additional file 3), although the outline of the cells revealed irregular borders and abnormal cell shapes. This suggests that the absence or mislocalization of E-cadherin is unlikely to be the reason for failure of compaction.

**Figure 5:**
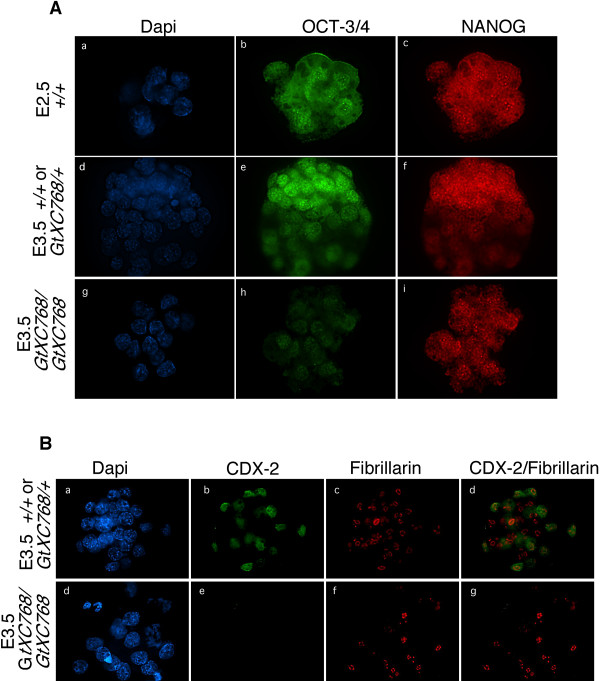
**Early lineage marker expression in *Rbm19*^*GtXC768*/*GtXC768 *^mutant embryos**. Embryos from an *Rbm19*^*GtXC768*/+ ^intercross were sorted based on appearance and designated either wild-type-appearing (*Rbm19*^*GtXC768*/+ ^or *Rbm19*^+/+^) or *Rbm19*^*GtXC768*/*GtXC768 *^mutants. (A) Double immunofluorescence staining for embryonic stem cell markers OCT-3/4 and NANOG reveals uniform expression in cleavage stage embryos, with progressive segregation of immunopositive cells to the inner cell mass in wild-type-appearing E3.5 embryos. In *Rbm19*^*GtXC768*/*GtXC768 *^embryos arrested at the 16-cell stage, the level of OCT-3/4 is diminished, but NANOG expression remains comparable to WT morula. There is no spatial segregation across the mutant embryos for either protein. (B) Double immunofluorescence staining for the trophectoderm marker CDX2 and the nucleolar protein Fibrillarin (positive control). In the wild-type-appearing embryos CDX2 expression was found in a subset of cells corresponding to the prospective trophectoderm, whereas in the *Rbm19*^*GtXC768*/*GtXC768 *^embryos there was no CDX2 expression detectable.

To assess whether *Rbm19*^*GtXC768*/*GtXC768 *^mutant embryos form the first embryonic lineages, we performed immunostaining for the lineage markers OCT-3/4, NANOG and CDX2 (Figure 5). Normally, OCT-3/4 and NANOG proteins are expressed in all the blastomeres of morula-stage embryos. Then, in concert with morula compaction, the outer blastomeres become specified to form the trophectoderm lineage. These cells gradually lose OCT-3/4 and NANOG expression and begin to express the trophectoderm marker CDX2. In the *Rbm19*^*GtXC768*/*GtXC768 *^mutants, OCT3/4 expression is substantially diminished, whereas NANOG expression remains similar to wild-type morulae. Notably, there was no CDX2 expression detectable in the mutants, indicating that developmental arrest occurs prior to trophectoderm specification.

### Apoptosis of blastomeres in *Rbm19*^*GtXC768/GtXC768 *^mutant embryos

**Figure 6:**
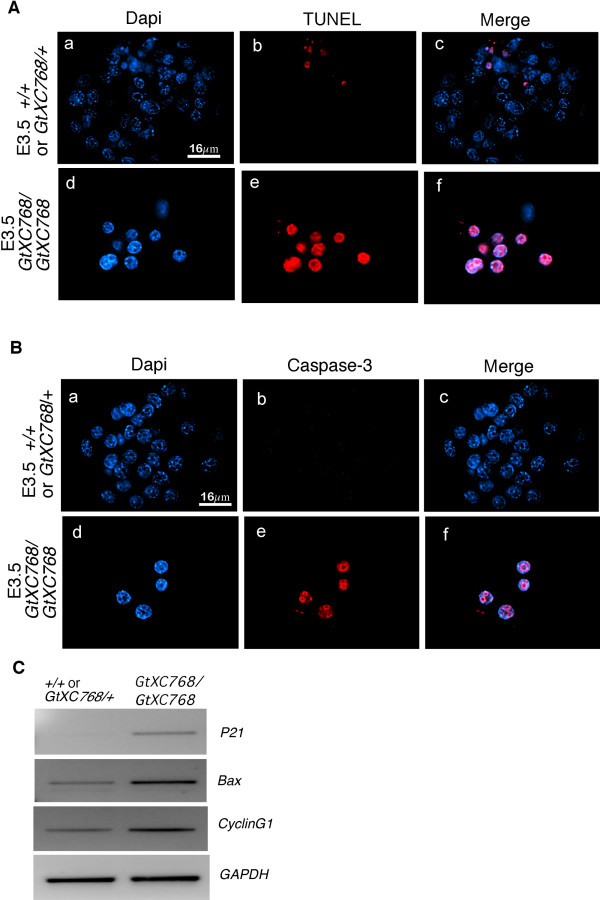
**Apoptosis and P53 activation in *Rbm19*^*GtXC768*/*GtXC768 *^mutant embryos**. Embryos from an *Rbm19*^*GtXC768*/+ ^intercross were sorted based on appearance and designated either wild-type-appearing (*Rbm19*^*GtXC768*/+ ^or *Rbm19*^+/+^) or *Rbm19*^*GtXC768*/*GtXC768 *^mutants. (A) Detection of apoptosis by in situ TUNEL assay. Wild type-appearing blastocysts exhibit very few TUNEL-positive cells, whereas extensive apoptosis was observed in the growth-arrested *Rbm19*^*GtXC768*/*GtXC768 *^embryos. (B) Cleaved caspase-3 staining of *Rbm19*^*GtXC768*^/^*GtXC768 *^embryos showed strong staining of most cells in mutant embryos, suggesting apoptotic cell death. (C) P53 target gene expression in wild type appearing and *Rbm19*^*GtXC768*^/^*GtXC768 *^mutant E3.5 embryos revealed by semi-quantitative RT-PCR. *p21*, *Bax *and *CyclinG1 *are upregulated in the mutant; *GAPDH *was used as a loading control.

To characterize the cellular process that leads to developmental arrest, we tested markers for apoptosis in the E3.5 *Rbm19*^*GtXC768*/*GtXC768*^mutant embryos. We performed both TUNEL assay and immunostaining for cleaved caspase 3, and found that about 90% of the mutant nuclei were positive for both markers of apoptosis (22/24 cells) (Figure 6A and 6B, respectively). In contrast, the wild-type embryos showed minimal staining for TUNEL and only background staining for caspase 3. The P53 tumor suppressor protein is a known inducer of apoptosis and has been shown to mediate the nucleolar stress response [22]. We tested the transcriptional activation of known P53 targets in the *Rbm19*^*GtXC768*/*GtXC768 *^mutant embryos by semi-quantitative RT-PCR (Figure 6C). We show that *p21*, *Bax*, and *cyclinG1 *are upregulated in the *Rbm19*^*GtXC768*/*GtXC768*^mutant embryos. Thus, both arms of the P53 effector pathway-cell cycle arrest and apoptosis-appear to be upregulated.

One possible reason for the timing of the *Rbm19*^*GtXC768*/*GtXC768*^mutant phenotype is the exhaustion of maternal gene product at the 8–16 cell stage, assuming that *Rbm19 *gene product is present prior to zygotic transcription. By RT-PCR using RNA from wild-type 2-cell stage embryos we detected *Rbm19 *expression (Additional file 4). Thus *Rbm19 *may be required even earlier than the 8–16 cells stage, since the early *Rbm19*^*GtXC768*/*GtXC768 *^mutant phenotype may be complemented by wild-type gene product from the heterozygote mother.

## Discussion

Here we report that a gene-trap insertion, resulting in disruption of the nucleolar protein RBM19, arrests embryonic development in the mouse prior to implantation. The mutant allele resulting from the gene trap may be partially functional, and maternal *Rbm19 *RNA is present in the 2-cell stage embryo, suggesting that RBM19 could be required even earlier than E3.5. In somatic cells RBM19 is a component of the nucleolus, and the timing of arrest (8–16 cell stage) coincides with the formation of the nucleolus. Also, localization of RBM19 changes from cytoplasm to nucleolus at the same stage as developmental arrest of the *Rbm19*^*GtXC768*/*GtXC768 *^mutant. The assembly of the nucleolus is a prerequisite for embryonic development [1]. Failure of nucleologenesis could trigger a nucleolar stress response that induces cell cycle arrest and/or apoptosis via activation of P53 [22] as one of many possible mechanisms to account for the *Rbm19*^*GtXC768*/*GtXC768*^mutant phenotype.

There are notable similarities and differences between RBM19 and other nucleolar proteins that have been studied by genetic inactivation in the mouse. Several others were found to be required at the morula stage, including fibrillarin [5], PES-1 [4], SURF6 [7], BYSL [23], and RNA polymerase 1–2[6]. These proteins all participate in various aspects of rRNA synthesis or processing. Thus impaired ribosome biogenesis may be the common molecular defect underlying these phenotypes. However, other genes that encode proteins essential for ribosome biogenesis do not cause morula arrest upon their mutation. For example, TIF1a is essential for rRNA transcription [24], and its ablation in the mouse leads to nucleolar disruption and p53-mediated apoptosis [8]. Yet, *Tif1a *mutant embryos survive to E9.5 (albeit 10-fold smaller than wild-type) and form rudiments of most organs. Similarly, ablation of the *B23 *gene results in embryonic lethality and genomic instability, but the embryos survive to E9.5-E12.5 [9]. Thus, a ribosome biogenesis defect cannot be predicted to uniformly induce early embryonic lethality. The reason for this may be that, while nucleologenesis and ribosome biogenesis are intimately linked, there may be differential effects on one and not the other, since the nucleolus has other roles as well. It may be difficult to sort out exactly which phenotypes are attributable to a shortfall in ribosome biogenesis and which are due to induction nucleolar stress signals. Since stress signals may be mediated in large by P53, analyzing the effects of nucleolar protein mutations in a *P53 *null background may provide some clues. For example, genetic inactivation of *P53 *mitigated, but did not completely rescue, the loss of B23 in the mouse [9]. We have generated *Rbm19*^*GtXC768*/*GtXC768 *^*/p53*^-/- ^double mutants and in preliminary studies it appears that loss of *p53 *does not rescue the *Rbm19 *mutant phenotype.

Nucleologenesis in the early embryo is first detectable upon activation of rRNA transcription on the surface of the NPB after the 2^*nd *^cell cycle [3]. The developmental events between the onset of rRNA transcription and nucleolar maturation are poorly understood. Nucleolar proteins studied to date localize to the surface of the nucleolar precursor body (NPB) [2]. We show that RBM19 protein does not conform to this localization pattern, but instead remains in the cytoplasm through the morula stage, then localizes to the nucleolus in the blastocyst. This would suggest that RBM19 plays a role the early embryo distinct from its previously characterized function in rRNA processing. One possible role might be coordinating other nucleolar proteins for transport to the nucleus, to insure proper nucleolar assembly [19].

Our finding that CDX2 expression is completely absent in *Rbm19*^*GtXC768*/*GtXC768 *^mutants identifies the first lineage specification event in the embryo as the stage at which developmental arrest occurs. It is tempting to speculate that the nucleolus plays a permissive role in regulating this developmental step. According to such a model, the developmental program may utilize the forming nucleolus as a sensor of normal development. If nucleologenesis is indeed determinative, failure of this process may represent one reason for arrest prior to implantation.

## Conclusion

The *Rbm19*^*GtXC768 *^mutation induces arrest of development as the nucleolus is maturing. Since nucleolar maturation occurs coincident with the stage-specific localization of RBM19 from the cytoplasm to the nucleolus, the underlying cause of arrest may be failure of nucleologenesis in concert with a P53-mediated stress response.

## Methods

### Insertional mutation of the Rbm19 locus

A mouse ES cell line (XC768) containing an insertional mutation of the *Rbm19 *locus was generated by BayGenomics [15]. The gene-trap vector used (pGT1Lxf) contains a splice-acceptor sequence upstream of the reporter gene βgeo (a fusion of β-galactosidase and neomycin phosphotransferase II) resulting in the generation of a truncated RBM19-β-geo fusion protein. ES cell line XC768 was injected into C57BL/6 blastocysts to generate chimeric mice, which were then intercrossed to establish the *Rbm19*^*GtXC768 *^mutant germline. The mice were weaned at 21 days of age, and housed in a barrier facility with a 12 h light/12 h dark cycle. All animal work was carried out in compliance with internationally recognized guidelines as per the Animal Welfare Act, under protocols approved by the MCW IACUC (protocol# 235-04-2).

### Genotype analysis

Mouse genomic tail DNA was prepared according to standard procedures. A duplex PCR strategy was developed to distinguish between the wild type and *Rbm19*^*GtXC768 *^mutant alleles. *Rbm19*-specific primers amplify a 296-bp DNA fragment across the insertion site, which is disrupted by the gene trap vector in the mutant. (*Rbm19*-forward: 5'-ATTGCCCTTGCTCACATTTC-3'. *Rbm19*-reverse: 5'-AGGCCTGACCACACTGACTT-3'.) β-*geo *primers amplify a 507-bp fragment from the gene trap vector. (β-geo-forward: 5'-CTGGCGTAATAGCGAAGAGG-3'. β-geo-reverse: (5'-CCGCCACATATCCTGATCTT-3').

### Blastocyst collection, culture in vitro, and genotyping

Blastocysts were collected at embryonic development day 3.5 (E3.5) by flushing the uterus with M2 (MR-015P-D, Chemicon). The blastocysts were transferred to KSOM (MR-020P-F, Chemicon), covered with mineral oil (M-5310, Sigma) and cultured at 37°C with 5% CO_2_. For genotyping, E3.5 embryos were collected into PCR tubes with 10 μL 1×PCR buffer (Qiagen), to which were added 1 μL 400 μg/ml proteinase K (#03115828001, Roche) incubated for 2 h at 50°C, followed by incubation at 98°C for 10 min to inactivate the proteinase K. The lysates were cleared by a 5 min centrifugation at 3000 rpmand used directly for PCR amplification.

### RNA preparation and RT-PCR

RNA isolation from E3.5 embryos was performed using the Nano-scale RNA Purification Kit (MPS04050, EPICENTRE Biotechnologies). Reverse transcription was carried out using a random hexamer primer. Gene-specific primers used are shown in Table 2. PCR products were separate on 2% agarose gels and visualized by staining with ethidium bromide.

**Table 2:** Primers used for semi-quantitative RT-PCR and conditions of PCR reaction

**Gene**	**Dir**	**Primer sequences (5'-3')**	**Product size**	**Annealing temp (°C)**
*P21*	F R	GTCCAATCCTGGTGATGTCC GTTTTCGGCCCTGAGATGT	410	55

*Bax*	F R	TGCAGAGGATGATTGCTGAC ACTCCAGCCACAAAGATGGT	313	55

*CyclinG1*	F R	AATGTTGCTGGGAAGTCAGG GCAAGGTGCTGAGGTTTCTC	352	55

*Rbm19*	F R	CGGAGCGAAGTAAGACTGTG CTCTGTGGTGCTGAAGTTG	470	53

*β*-geo	R	AGT ATC GGC CTC AGG AAG ATC G		53

*GAPDH*	F R	ATTGTCAGCAATGCATCCTG TTCAGCTCTGGGATGACCTTGCC	243	59

### Immunofluorescence Studies and TUNEL Assay

For immunofluorescence analysis, preimplantation embryos at the indicated stages were incubated for 1 minute in acid Tyrodes solution (Specialty Media, MR-004-D, Chemicon) to dissolve the zona pellucida, then fixed in PBS/3.2% PFA for 5 minutes. After washing briefly in PBS/10 mM glycine, embryos were permeablized for 3 minutes in 0.5% Nonidet P-40 (in 1% BSA PBS), then blocked in PBS/3.0%BSA for 1 hour at room temperature, incubated with primary antibodies at 4°C overnight, and then washed in block solution and incubated with appropriate secondary antibodies (Molecular Probes or Jackson Immunoresearch) diluted 1:500, and counterstained with DAPI (0.1 μg/ml) for 5 min at room temperature. Immunostained embryos were placed on microscope slides with Gel/Mount (#M01, Biomeda) and covered with glass cover slips. Fluorescence was captured using a mechanized Zeiss Axioplan II microscope driven by OpenLab (Improvision) to generate z-stacks (0.2 μm steps). Z-stacks were deconvolved using Volocity 3.0 (Improvision) via an iterative algorithm. Apoptosis was detected by the TdT-mediated dUTP-fluorescein nick end-labeling (TUNEL) assay by using the In Situ Cell Death Detection kit (Cat# 12 156 792 910, Roche). In all cases, embryos shown are representative of at least 3 independent experiments, in which about 10 embryos (7–8 normal and 2–3 mutant) were stained and imaged.

For the Br-UTP incorporation assays, permeablization was performed with 100 μg/mL Saponin (Sigma, S4521-10G) in KSOM for 5 minutes at room temperature, and then washed with KSOM. Embryos were incubated with KSOM supplemented with 200 μM Br-UTP for 60 minutes at 37°C. After washing with KSOM, embryos were fixed in 3.2% PFA/PBS for 5 minutes at room temperature. RNA synthesis was monitored with anti-Br-dUTP antibody (1:100, Sigma).

### Real-time Quantitative PCR

The number of copies of the gene trap vector was determined by comparing the Cycle Threshold (Ct) between *Rbm19 *and β-geo in heterozygote mice. PCR primers used for genotyping were used to generate PCR products from heterozygote mouse DNA. These were gel-purified and quantified, and used to generate a standard curve for each primer pair. Ct was measured using SYBR green and the Biorad/MJ Research Opticon2.

### Western blot analysis

Total mouse duodenum lysates were fractionated using sodium dodecyl sulfate-polyacrylamide gel electrophoresis and transferred onto a polyvinylidene difluoride membrane (Millipore Corporation). After blocking with 5% nonfat dry milk (Bio-Rad Laboratories, Inc., Hercules, CA) for 3 h at room temperature, blots were incubated with a 1:500 dilution of RBM19 antibody [14] at 4°C overnight, then incubated with 1:8000 horseradish peroxidase-conjugated anti-rabbit secondary antibody and developed using the ECL system (Pierce Biotechnology Inc., Rockford, IL).

### Electron Microscopy

E3.5 embryos were fixed in 2% glutaraldehyde. After dehydration, embryos were embedded in epoxy resin. Sections were contrasted with uranyl acetate and lead citrate and examined using a Hitachi 600 electron microscope.

### Primary antibodies

Anti-B23/nucleophosmin (cat# 32-5200, Zymed), anti-GW182 (cat# ab15843, Abcam), ribosomal protein S6 (cat# 54D2 Cell Signaling), anti-fibrillarin (cat# ab5821, Abcam), anti-Cleaved Caspase-3 (cat# 9661, Cell Signaling), anti-nucleostemin (cat# AF1638, R&D systems).

## Abbreviations

RBM19: RNA-binding motif protein 19; NPB: nucleolar precursorbody; NORs: nucleolar organizing regions; RRM: RNA recognition motifs; β-geo: a fusion of β-galactosidase and neomycin phosphotransferase II; TUNEL: TdT-mediated dUTP-fluorescein nickend-labeling; PES-1: pescadillo-1; (kD: kilodaltons.

## Authors' contributions

JZ performed the experiments, analyzed the data, and edited the manuscript. AJT performed RNA extraction, quantitative PCR, and analyzed the data. ANM designed the study, analyzed the data and wrote the manuscript.

## Supplementary Material

Additional file 1 **RBM19 and GW182 do not colocalize in the cytoplasm of 8-cell stage mice embryos**. (A) Double staining of RBM19 (red) and GW182 (green), counterstained with DAPI. (B) Enlargement of the box in panel A. The arrow shows GW182 positive foci in the cytoplasm. (C) RBM19 immunofluorescence showing cytoplasmic foci (D) Merged image showing disRBM19 and GW182 do not colocalize. Click here for file

Additional file 2 **In situ assay for ribosomal RNA transcription**. (A-D) E2.5 morula embryos derived from wild type mice were cultured in the presence of BrUTP and then immunostained to detect label incorporation. Panels show colocalization of BrUTP and Fibrillarin. (E-L) Embryos from a *Rbm19*^*GtXC768*/+ ^intercross were sorted based on appearance and designated either wild-type-appearing (*Rbm19*^*GtXC768*/+ ^or *Rbm19*^+/+^) or *Rbm19*^*GtXC768*/*GtXC768*^mutants. (E-H) E3.5 *Rbm19*^*XC*768/+*or*+/+ ^were labeled with Br-UTP while cultured in vitro. Circles indicate colocalization of Br-UTP-labeled RNA (green) with the fibrillarin (red). (I-L) *Rbm19*^*XC*768/*XC*768 ^mutant embryos incorporated BrUTP in nucleoli comparable to wild-type embryos. Click here for file

Additional file 3 **E-cadherin localization in *Rbm19 *mutant embryos**. E-cadherin antibodies were used to stain E3.5 embryos from a *Rbm19*^*XC*768/+ ^in-cross. The surface cells of WT blastocyst-stage embryos were imaged (left panel), showing polygonal cells with staining at cell borders. The *Rbm19*^*XC*768/*XC*768 ^mutants remained in an uncompacted 8–16 cell stage, yet E-cadherin staining was evident at the cell borders. The cells were larger with more irregular shapes, suggesting failure of epithelial organization in the prospective trophectoderm. Click here for file

Additional file 4 **RT-PCR from 2-cell stage embryos showing maternal expression of *****Rbm19***. Embryos flushed from the oviducts of mice 1 day after appearance of the vaginal plug were at the 2-cell stage. RNA isolated from ten 2-cell stage embryos (CD-1 strain) was subjected to reverse transcription and PCR using primers shown in Table 2. Agarose gel shows a band at about 500 bp (arrow), the expected size for the *Rbm19*-specific product. This demonstrates that *Rbm19 *RNA is deposited in the egg prior to fertilization. Click here for file
