# Supplementary figures and images for: RBM19 is essential for preimplantation development in the mouse
Source: BMC Dev Biol. 2008 Dec 16;8:115. doi: 10.1186/1471-213X-8-115 (PMC2627835; doi:10.1186/1471-213X-8-115)

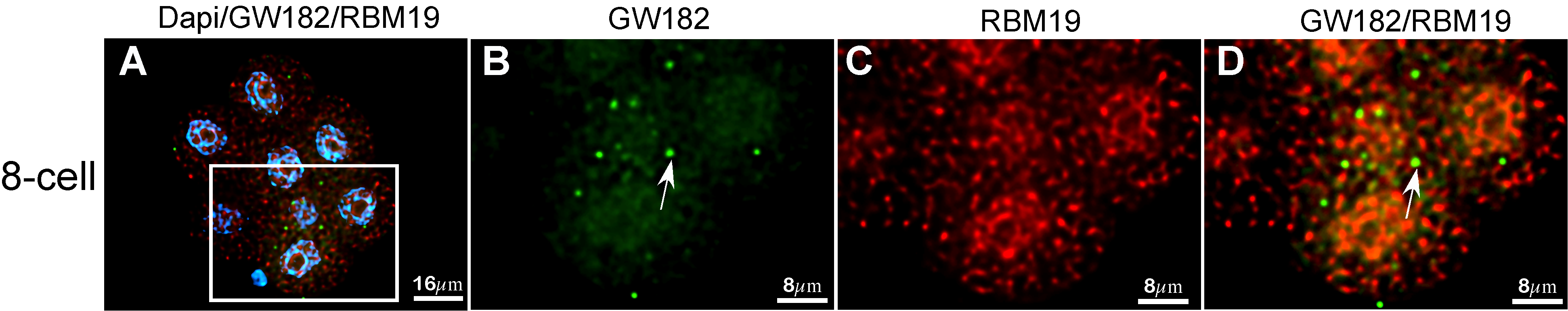

Supplement: Additional file 1 — RBM19 and GW182 do not colocalize in the cytoplasm of 8-cell stage mice embryos. (A) Double staining of RBM19 (red) and GW182 (green), counterstained with DAPI. (B) Enlargement of the box in panel A. The arrow shows GW182 positive foci in the cytoplasm. (C) RBM19 immunofluorescence showing cytoplasmic foci (D) Merged image showing disRBM19 and GW182 do not colocalize. [file 1471-213X-8-115-S1.tiff]

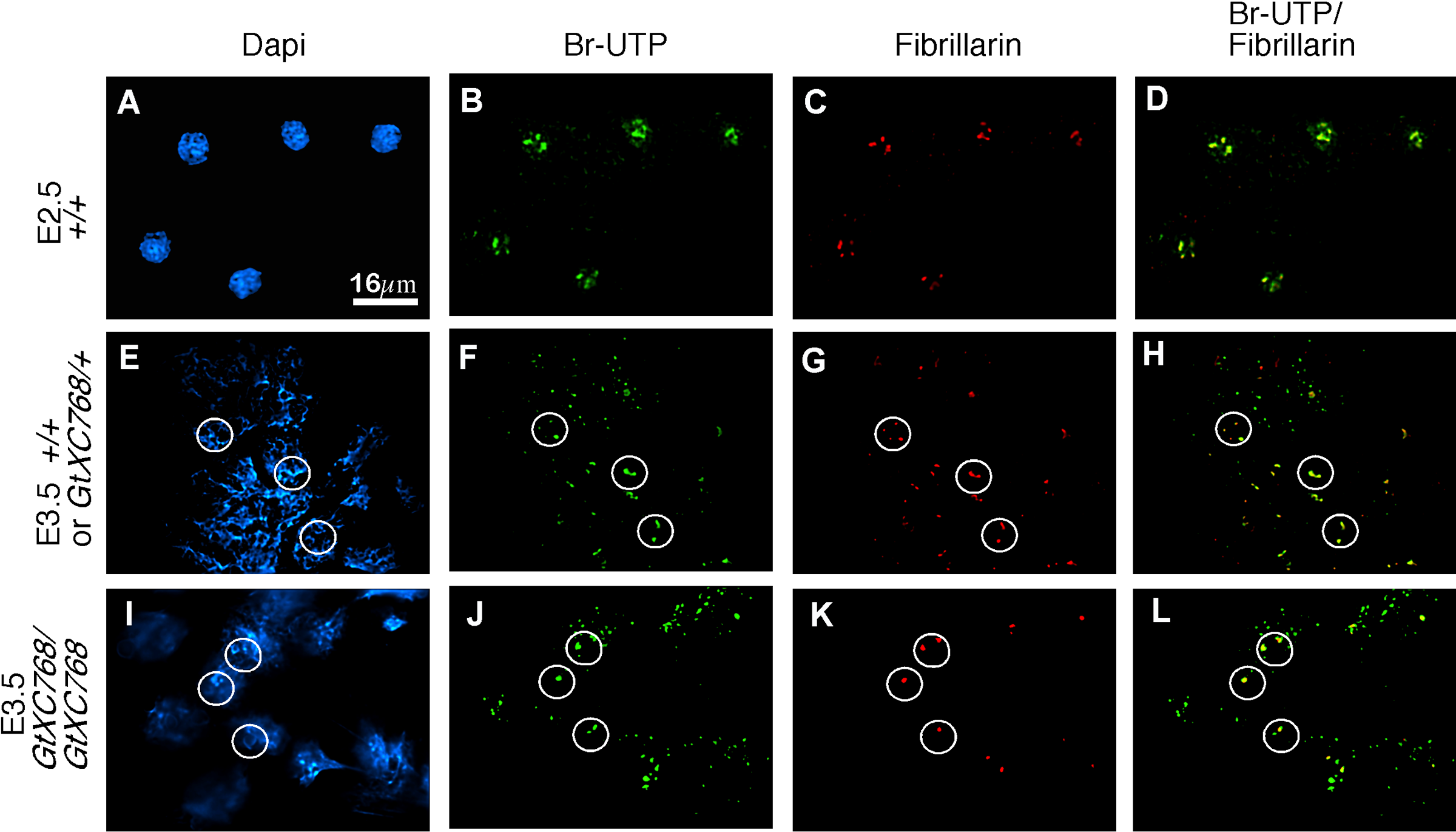

Supplement: Additional file 2 — In situ assay for ribosomal RNA transcription. (A-D) E2.5 morula embryos derived from wild type mice were cultured in the presence of BrUTP and then immunostained to detect label incorporation. Panels show colocalization of BrUTP and Fibrillarin. (E-L) Embryos from a Rbm19GtXC768/+ intercross were sorted based on appearance and designated either wild-type-appearing (Rbm19GtXC768/+ or Rbm19+/+) or Rbm19GtXC768/GtXC768mutants. (E-H) E3.5 Rbm19XC768/+or+/+ were labeled with Br-UTP while cultured in vitro. Circles indicate colocalization of Br-UTP-labeled RNA (green) with the fibrillarin (red). (I-L) Rbm19XC768/XC768 mutant embryos incorporated BrUTP in nucleoli comparable to wild-type embryos. [file 1471-213X-8-115-S2.tiff]

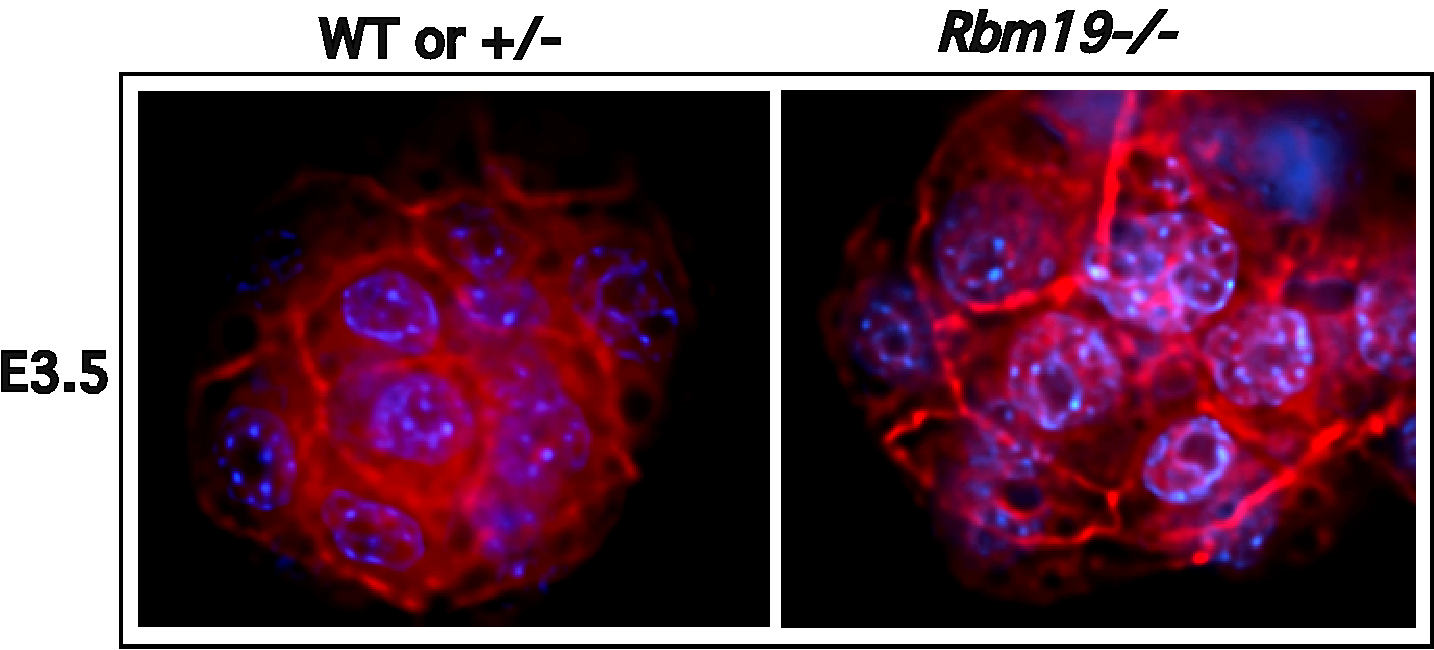

Supplement: Additional file 3 — E-cadherin localization in Rbm19 mutant embryos. E-cadherin antibodies were used to stain E3.5 embryos from a Rbm19XC768/+ in-cross. The surface cells of WT blastocyst-stage embryos were imaged (left panel), showing polygonal cells with staining at cell borders. The Rbm19XC768/XC768 mutants remained in an uncompacted 8–16 cell stage, yet E-cadherin staining was evident at the cell borders. The cells were larger with more irregular shapes, suggesting failure of epithelial organization in the prospective trophectoderm. [file 1471-213X-8-115-S3.tiff]

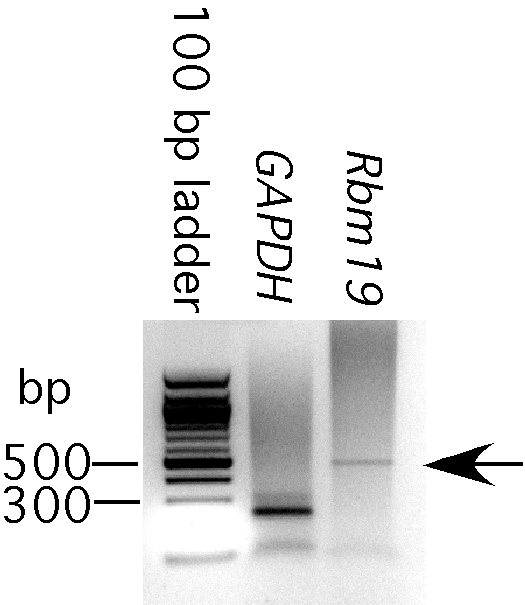

Supplement: Additional file 4 — RT-PCR from 2-cell stage embryos showing maternal expression of Rbm19. Embryos flushed from the oviducts of mice 1 day after appearance of the vaginal plug were at the 2-cell stage. RNA isolated from ten 2-cell stage embryos (CD-1 strain) was subjected to reverse transcription and PCR using primers shown in Table 2. Agarose gel shows a band at about 500 bp (arrow), the expected size for the Rbm19-specific product. This demonstrates that Rbm19 RNA is deposited in the egg prior to fertilization. [file 1471-213X-8-115-S4.tiff]
